# *Helicobacter pylori* infection and Parkinson’s Disease: etiology, pathogenesis and levodopa bioavailability

**DOI:** 10.1186/s12979-023-00404-1

**Published:** 2024-01-02

**Authors:** Bang-rong Wei, Yu-jia Zhao, Yu-feng Cheng, Chun Huang, Feng Zhang

**Affiliations:** 1 Key Laboratory of Basic Pharmacology of Ministry of Education and Joint International Research Laboratory of Ethnomedicine of Ministry of Education and Key Laboratory of Basic Pharmacology of Guizhou Province and Laboratory Animal Centre, Zunyi Medical University, Zunyi, Guizhou China; 2 The Fifth People’s Hospital of Chongqing, Chongqing, China

**Keywords:** Parkinson’s disease, *Helicobacter pylori*, Levodopa, Lipopolysaccharide, Vacuolar cytotoxin A

## Abstract

Parkinson’s disease (PD), a neurodegenerative disorder with an unknown etiology, is primarily characterized by the degeneration of dopamine (DA) neurons. The prevalence of PD has experienced a significant surge in recent years. The unidentified etiology poses limitations to the development of effective therapeutic interventions for this condition. *Helicobacter pylori* (*H. pylori*) infection has affected approximately half of the global population. Mounting evidences suggest that *H. pylori* infection plays an important role in PD through various mechanisms. The autotoxin produced by *H. pylori* induces pro-inflammatory cytokines release, thereby facilitating the occurrence of central inflammation that leads to neuronal damage. Simultaneously, *H. pylori* disrupts the equilibrium of gastrointestinal microbiota with an overgrowth of bacteria in the small intestinal known as small intestinal bacterial overgrowth (SIBO). This dysbiosis of the gut flora influences the central nervous system (CNS) through microbiome-gut-brain axis. Moreover, SIBO hampers levodopa absorption and affects its therapeutic efficacy in the treatment of PD. Also, *H. pylori* promotes the production of defensins to regulate the permeability of the blood-brain barrier, facilitating the entry of harmful factors into the CNS. In addition, *H. pylori* has been found to induce gastroparesis, resulting in a prolonged transit time for levodopa to reach the small intestine. *H. pylori* may exploit levodopa to facilitate its own growth and proliferation, or it can inflict damage to the gastrointestinal mucosa, leading to gastrointestinal ulcers and impeding levodopa absorption. Here, this review focused on the role of *H. pylori* infection in PD from etiology, pathogenesis to levodopa bioavailability.

## Introduction

Parkinson’s disease (PD) is the second most prevalent neurodegenerative disorder, following Alzheimer’s disease (AD) [1]. It is projected that by 2040, the global PD population will exceed 14 million individuals [2]. Numerous studies have demonstrated that the older adult has a higher susceptibility to PD [3]. PD is characterized by a progressive degeneration of neurons in the brain, leading to their eventual demise and disruption of normal dopamine (DA) neuronal activity [4]. Notably, two prominent neuropathological manifestations of PD are the death of nigrostriatal DA neurons and the abnormal aggregation of α-synuclein (α-syn) with the formation of Lewy vesicles [5]. The clinical symptoms of PD can be categorized into motor and non-motor symptoms. Motor symptoms mainly encompass dyskinesia, tremor, rigidity and postural instability [6], whereas non-motor symptoms predominantly consist of depression, anxiety, sensory disturbances, sleep disturbances, gastrointestinal dysfunction, amnesia, olfactory loss, and dementia [7, 8]. Previous studies indicated that non-motor symptoms manifested earlier than motor symptoms [9]. Moreover, PD patients frequently reported experiencing gastrointestinal disorders prior to the onset of motor symptoms. Gastrointestinal symptoms in PD have a significantly high prevalence globally [7]. Typically, the symptoms of PD exhibit a gradual deterioration as the disease progresses [6]. Regrettably, there is currently no medical intervention capable of completely eradicating PD. Clinical management of PD primarily relies on the administration of levodopa to alleviate motor symptoms [10].

*Helicobacter pylori* (*H. pylori*), a Gram-negative, spiral-shaped, microaerophilic and flagellated bacterium, is widely prevalent worldwide, infecting approximately half of the global population [11, 12]. It predominantly colonizes the gastric mucosa in humans and predisposes individuals to various gastric mucosal diseases, including gastric ulcers and gastric cancer [11]. *H. pylori* can enter the brain via two distinct pathways: the oral-nasal olfactory pathway and the gastrointestinal tract associated retrograde axonal transport pathway [13]. Monocytes infected with *H. pylori* traverse the blood-brain barrier (BBB), which is compromised due to chronic infection and the release of pro-inflammatory cytokines. Consequently, this process leads to the occurrence of neurodegeneration [14]. Extensive evidences have demonstrated the association between *H. pylori* and various central nervous system (CNS) disorders, such as depression, anxiety, PD, AD, Multiple sclerosis (MS), Green-Barré syndrome (GBS), and Bickerstaff brainstem encephalitis (BBE), among others [13, 15]. The mechanisms by which *H. pylori* contributes to PD are believed to involve the production of toxins, disruption of the delicate equilibrium of intestinal microbes, stimulation of pro-inflammatory cytokines production, and interference with the effectiveness of PD medications [16, 17]. This review aimed to summarize the association between *H. pylori* infection and PD, focusing on the etiology, pathogenesis, and levodopa bioavailability, to provide new insights into the treatment of PD.

## Microbiome-gut-brain axis: *H. Pylori* infection and PD

*H. pylori* infection has been observed to disrupt the delicate equilibrium of the microbiota within the gastrointestinal tract [18]. It is widely acknowledged that the maintenance of human health heavily depends on the dynamic balance of the gastrointestinal microbiota [19]. When this equilibrium is disrupted, the body becomes vulnerable to various associated diseases [20]. *H. pylori* primarily influences the intestinal microbiota by means of virulence factors, such as cytotoxin-related gene A (CagA), vacuolar cytotoxin A (VacA), urease (Ure), arginase (Arg), lipopolysaccharide (LPS), and neutrophil activating protein (NAP) [11]. This phenomenon involves the fascinating concept of the microbiome-gut-brain axis, which encompasses various signaling pathways, including neuronal, metabolic, endocrine, and immune pathways, through which the gut microbiome communicates with the brain [15, 21]. Importantly, the gut microbiota can up-regulate inflammation both locally and systemically [21]. Dysregulation of the intestinal ecological and heightened intestinal permeability leads to overstimulation of the innate immune system, inducing systemic and CNS inflammation [21]. In addition, *H. pylori* has the potential to indirectly affect the microbiome-gut-brain axis through its influence on the intestinal microbiota, consequently resulting in the development of CNS disorders [13].

**Fig. 1:**
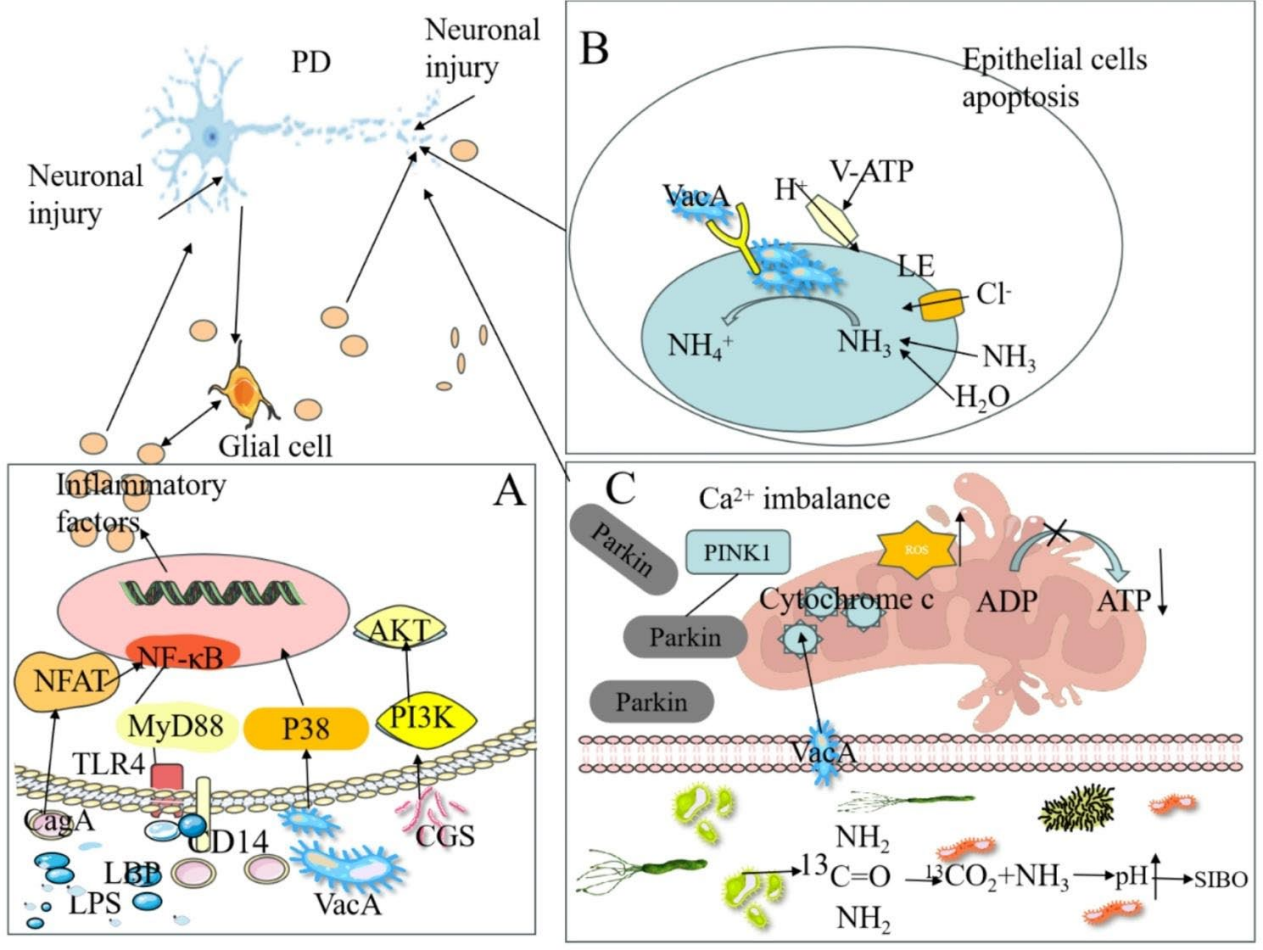
Potential mechanisms of the association of *H. pylori* infection with PD. (**A**) *H. pylori* virulence factors, such as LPS, CagA, VacA, and CGs, activate NF-kB or PI3K-AKT pathway to induce the production of pro-inflammatory factors of the T cells, which further stimulate glial cells, leading to neuronal injury. (**B**) VacA monomers assemble into oligomers and then translocate to the membranes of LEs, facilitating the entry of chloride ions into the lumen, which in turn enhances the activity of v-ATPase proton pump and reduces the intraluminal pH. Additionally, VacA induces apoptosis of epithelial cells that leads to “leaky gut” and increases the permeability of the blood-brain barrier, eventually impact nerve cells. (**C**) *H. pylori* infection cause SIBO, leading to the impairment of mitochondrial function and subsequent hindrance of ATP production and calcium influx, which are crucial for maintaining neuronal activity. Moreover, its virulence factor, VacA, can enter mitochondria and stimulate the release of cytochrome c from these organelles, further leading to mitochondrial impairment. Abbreviations: PD: Parkinson’s disease; CagA: Cytotoxin-related gene A; VacA: Vacuolar cytotoxin A; Ure: Urease; LPS: Lipopolysaccharide; TLR4: Toll-like receptor 4; LBP: Lipopolysaccharide binding protein; CG: Cholesterol glucosides; LEs: Late endonuclear bodies

Abnormal folding and aggregation of α-syn in the enteric nervous system (ENS) of PD patients causes the formation Lewy bodies and neuritis [22]. Furthermore, Lewy bodies are discerned in the gastrointestinal tract of numerous PD patients who do not exhibit PD symptoms [23]. These discoveries have fostered an increasing recognition that the origin of PD might lie within the intestine. Consequently, a hypothesis has been put forth suggesting that the misfolding and aggregation of α-syn initially occurs in the intestine and subsequently spreads to brain via the vagus nerve [24]. This hypothesis was expeditiously validated through the administration of human brain PD lysate containing α-syn into the intestinal wall of rats, which resulted in the rapid transportation of α-syn via the vagus nerve into the brainstem, specifically in the dorsal motor nucleus of the vagus nerve [25]. The pivotal role of the vagus nerve in this process becomes evident. As one of the largest nerves connecting the gastrointestinal tract to the brain, the vagus nerve has been observed to diminish the risk of PD when subjected to vagotomy, especially for full truncal vagotomy [24]. This substantiates the notion that PD and the gut are inextricably linked [26]. On the other hand, it has been demonstrated that a significant proportion of the impacts exerted by the gut microbiota on brain function are contingent upon vagal activation. This finding serves to reinforce the notion that the gastrointestinal tract and CNS could establish a reciprocal relationship via the brain-gut axis. At the beginning of the 20th century, the association between infection and PD was widely regarded as highly consequential [27]. Since then, increasing evidence focused their attention towards investigating the correlation between viral and bacterial infections and PD. Subsequently, in 1960, the association of *H. pylori* infection and PD was initially discovered [28]. Moreover, various studies have reported a prevalence range of 26.4–87.9% for *H. pylori* infection in PD patients. These patients were older and had worse motor function, suggesting a potential association between *H. pylori* infection and PD pathophysiology (Table 1). Additionally, the effect of *H. pylori* infection on PD motor results varied according to age [40]. Various viewpoints exist regarding the factors that influence PD pathogenesis upon *H. pylori* infection, with particular emphasis placed on toxin production by *H. pylori* (Fig. 1A), disruption of intestinal flora, release of massive pro-inflammatory cytokines, activation of microglia and astrocyte, and dysfunction of BBB [17].

**Table 1:** Prevalence of *H. pylori* infection in patients with PD

References	Mean age (Years)	Prevalence of *H. pylori* infection (%)	*H. pylori* infection (n)	PD patients (n)
Dobbs, et al. 2012 [29]	65.0	70.5	36	51
Rahne, et al. 2013 [30]	72.1	26.7	20	75
Hashim, et al. 2014 [31]	65.1	32.9	27	82
Tan, et al. 2015 [32]	65.3	32.4	33	102
Efthyniou, et al. 2017 [33]	68.7	26.4	14	53
Liu, et al. 2017 [34]	63.4	45.8	22	48
Mridula, et al. 2017 [35]	60.0	50.0	18	36
Roshan, et al. 2019 [36]	70.5	66.7	33	99
Görgün, et al. 2019 [37]	68.7	87.9	83	73
Tan, et al. 2020 [38]	66.0	47.8	32	67
Lolekha, et al. 2021 [39]	63.6	55.0	22	40

## *H. Pylori* infection in PD pathogenesis

### *H. Pylori* virulence factors and PD

#### LPS

LPS, a group of phosphorylated lipoglycans, is primarily found in the outer membrane of Gram-negative bacteria [41]. The influence of *H. pylori* on the pathogenesis of PD has been reported to occur through the production of LPS [42, 43]. The detrimental effects of LPS on DA neurons are mainly attributed to its ability to trigger an inflammatory response [42]. This hypothesis was supported by a mouse model experiment conducted in 2017 and 2022, wherein the intraperitoneal injection of LPS elicited a robust pro-inflammatory response in mice, leading to the loss of nigrostriatal DA neurons [44, 45]. LPS treatment was administered subsequent to the successful establishment of the mouse model, and the cessation of LPS-induced damage to DA neurons was observed [45]. Moreover, LPS activates immune cells, such as T cells, in the gut and substantia nigra, thereby triggering a systemic inflammatory response by interacting with Toll-like receptor 4 (TLR4) located on the gut surface [46–48]. Among the entire TLR family, TLR4 exhibits a distinctive ability to recognize LPS derived from Gram-negative bacterial [48]. The process of LPS attachment involves the cluster of differentiation (CD14) in two forms [49]. Specifically, LPS is presented to membrane CD14 and TLR4 via the mediation of lipopolysaccharide binding protein (LBP). Additionally, it has been demonstrated that soluble CD14 and LBP play crucial roles in activating TLR upon LPS stimulation [50–52]. The activation of TLR4 by LPS can initiate various downstream pathways, including myeloid differentiation primary response 88 (MyD88) and NF-κB, which play critical roles in production of multiple pro-inflammatory cytokines (e.g., TNF-α, IL-1β, and IL-6) and oxidative stress molecules that contribute to neuroinflammation [53–55].

The pathology of PD is characterized by the misfolding and aggregation of α-syn, which activate microglia and astrocytes in the nigrostriatal region [6, 56, 57]. Lipid structures present in LPS induce the aggregation of α-syn [26, 58], and these lipid structures are mainly located in the outer membrane of Gram-negative bacteria. Furtherly, nuclear magnetic resonance (NMR) semi-quantitative analysis demonstrates that intact helical α-syn structures adopt a specific helical conformation in order to bind to the lipid surface [58, 59]. There are substantial evidences supporting this theory. In addition, α-syn monomers exhibited intense thioflavin T fluorescence after incubation with LPS [59]. When these α-syn protofibrils were injected into the mouse striatum, phosphorylated α-syn was observed throughout various regions of the brain including the striatum, substantia nigra, amygdala, and auditory cortex. This finding suggests that α-syn protofibrils induced by LPS possess toxic properties [58]. The neurotoxicity of these protofibrils is related to their structural and post-translational modifications, such as phosphorylation and nitration at Ser129 [59–61]. Also, LPS regulates α-syn in a concentration-dependent manner [58, 60]. Meanwhile, the presence of these pathological proteins hinders normal physiological functions of α-syn, while also exerting toxicity as an environmental stressor, thereby promoting inflammation, oxidative stress, and disruption of other physiological processes [62, 63]. Furthermore, this cascade of events triggers the release of pro-inflammatory and neurotoxic molecules by immune cells in the brain, ultimately leading to chronic neuroinflammation and neuronal death [64].

Besides, the induction of PD by LPS involves in the activation of glial cells, which in turn produce various stimulatory and regulatory factors [65, 66]. One notable effect is the excessive production of nitric oxide (NO) by microglia, which leads to DA neurodegeneration. Meanwhile, LPS up-regulates the expression of inducible nitric oxide synthase (iNOS) to promote NO release from microglia and astrocytes [64]. In addition, LPS can influence the activation of nicotinamide adenine dinucleotide phosphate (NADPH) oxidase enzymes, resulting in the production of reactive oxygen species (ROS) in microglia [67]. Interestingly, these ROS released from microglia not only directly damage DA neurons, but also up-regulate the expression of TNF-α [68], IL-1β and cyclooxygenase-2 (COX-2), leading to enhanced collective inflammatory damage to DA neurons [69, 70]. Among various cytokines released by LPS-activated glial cells, the pro-inflammatory factors, such as IL-1β and TNF-α, are believed to be the primary cytokines involved in LPS-induced DA neurodegeneration [69–71]. Furthermore, TNF-α increases the surface expression of tumor necrosis factor receptor 1 (TNFR1), thereby exacerbating LSP-induced DA neurotoxicity [64].

#### Cholesterol glucoside

*H. pylori* infection leads to the production of cholesterol glucosides (CGs) that have a detrimental effect on nerve cells, thereby initiating the degeneration of neurons and ultimately triggering PD [72]. CGs, which are lipid components of *H. pylori*, play a crucial role in enabling it to adapt to environmental changes. The synthesis of CGs necessitates the presence of cholesterol, which *H. pylori* cannot produce on its own and therefore relies on the consumption of host cholesterol [73]. Enzyme responsible to produce CGs is cholesterol-based glycosyltransferase (CGT), which is encoded by the *HP0421* gene [74]. This gene is required for *H. pylori* evasion of host immune cells [75]. AKT, a crucial protein kinase with anti-apoptotic properties, is believed to exert a protective function in neuronal injury by phosphorylating and inhibiting downstream regulators of apoptosis [76, 72]. Activation of AKT occurs in response to oxidative and hypoxia, enabling it to effectively process various types of stressful stimuli [77, 72]. The cytoprotective effect associated with AKT is abolished when its upstream activator, PI3K, is blocked, indicating the significance of the PI3K/AKT pathway in promoting cell survival [72]. This notion is further supported by the elimination of cytoprotective effect, when PI3K is inhibited during CG preconditioning [72]. The AKT pathway plays an important role in inhibiting cell death induced by various cytotoxic stress stimuli [77, 78]. In addition, impairment of AKT signaling has been implicated in neuronal death observed in various neurodegenerative disease animal models [72]. For instance, the absence of AKT signaling was identified as a potential contributor to cell death in motor neurons lacking adequate nutritional support, and interference with the PI3K/AKT pathway by CGs in T cells was implicated in the pathogenesis of PD [72, 79].

#### Cytotoxin

The cytotoxins, CagA and VacA, are considered as the main pathogenic factors in *H. pylori* infection [80]. CagA, a virulence factor of *H. pylori*, can be translocated by the type IV secretion system (T4SS) after attaching to cells, then activate T cells that result in chronic inflammation of gastric epithelial cells [81, 82]. It has the ability to activate transcription factor, NFAT, independent of its phosphorylation [82, 83]. Lipid rafts are cholesterol-rich membrane microstructural domains that provide entry points for many bacterial pathogens or their virulence factors. *H. pylori* utilizes host externalized phosphatidylserine (PS) to deliver CagA via T4SS [84], and CGT participates in this process by promoting *H. pylori* adhesion to host cells in a raft-dependent manner [68, 85].

VacA possesses the ability to form anion-selective channels within planar lipid bilayers, allowing for the conduction of chloride, bicarbonate, and organic molecules. This ability to stimulate intracellular acidic vesicle formation and disrupt cellular homeostasis ultimately leads to apoptosis (Fig. 1B) [74, 84, 86]. VacA monomers assemble into oligomers and then translocate to the membranes of late endonuclear bodies (LEs), where they form an anion-selective channel, enabling the entry of chloride ion into the lumen [87, 88]. This process subsequently enhances the activity of the v-ATPase proton pump and reduces the intraluminal pH [74]. Weak bases that can pass through the membrane diffuse into the LEs, in which they are protonated and trapped in an acidic environment [74]. LEs develops osmotic swelling, leading to cellular vacuolation [89, 90]. Additionally, VacA increases the permeability of the cell membrane, allowing for the influx of various anions and small molecules, such as chloride, urea and bicarbonate, into the extracellular space [91]. VacA induces vacuolation and apoptosis of epithelial cells, inducing disruption of tight junction integrity in the epithelium and resulting in increased permeability (“leaky gut”). This increased permeability allows various toxins to cross BBB and enter the brain, where they can impact nerve cells [74].

VacA activates p38MAPK in T cells, a crucial regulator of multiple cellular stresses. The activated form of p38MAPK then regulates several downstream transcription factors, including NF-κB, which up-regulates inflammatory molecules, such as COX-2 and iNOS, and promotes the development and progression of inflammatory response [74, 92, 93]. The p38MAPK signaling pathway participates in the neuroinflammatory responses mediated by microglia and astrocytes [94]. Activation of the p38 signaling pathway by VacA also induces the activation of activating transcription factor 2 (ATF-2) [95]. Furthermore, VacA can modulate the function of a variety of immune cells, including lymphocytes, macrophages, eosinophils, mast cells, and dendritic cells. In B cells, VacA has been observed to disrupt antigen presentation [18]. In macrophages, VacA contributes to the formation of macrovesicular bodies and hinders the maturation and functionality of vesicle compartments [96]. In addition, VacA modulates various signaling pathways in macrophages and induces apoptosis in these cells [97–99]. Consequently, these VacA-mediated effects might compromise the phagocytic ability of macrophages towards *H. pylori* [87], thereby exacerbating the effects of *H. pylori* on PD.

### *H. Pylori*, small intestinal bacterial overgrowth and PD

An abundance of bacteria in the small intestine, resulting in gastrointestinal symptoms, is referred to as small intestinal bacterial overgrowth (SIBO) [100]. It is widely acknowledged that constipation, a representative non-motor symptom of PD, exhibits a high prevalence. In 2021, a connection between SIBO and PD was first established through the presence of constipation, suggesting that the severity of constipation might increase the likelihood of SIBO [101]. In addition, elevated gastric pH and gastric mucosal atrophy could contribute to the development of SIBO [102]. *H. pylori* is capable of hydrolyzing urea to produce ammonia and carbonic acid in the stomach, where ammonia by-products serve to buffer gastric acid, causing an increase in gastric pH and the development of gastric mucosal atrophy. Moreover, proton pump inhibitors (PPIs), which are commonly used to treat *H. pylori* infection, further elevate gastric pH. Antibiotics utilized in the treatment of *H. pylori* infection disrupt the balance of intestinal flora, potentially leading to SIBO [102, 103]. In 2017 and 2018, two studies were conducted to investigate the relationship between SIBO and *H. pylori*. The pilot study conducted in 2017 examined a cohort of 109 patients diagnosed with both *H. pylori* infection and SIBO, revealing that 52.8% patients with *H. pylori* infection also presented with SIBO [104]. Conversely, only 21.9% patients without *H. pylori* infection met the criteria for SIBO [103]. These data suggested that the incidence of SIBO was twice as high in patients infected with *H. pylori* compared to those uninfected patient [105]. This conclusion was further supported by a separate study conducted in 2018, which reported that 53% patients with concurrent *H. pylori* infection and SIBO were identified [106].

It is noteworthy that *H. pylori* infection causes mitochondria impairment, and the coexistence of *H. pylori* and damaged mitochondria synergistically contribute to the progression of SIBO [107, 105] (Fig. 1C). Its virulence factor, VacA, can enter mitochondria and stimulate the release of cytochrome c from these organelles, further leading to mitochondrial impairment [104, 108, 109]. The emergence of SIBO further exacerbates mitochondrial dysfunction, and chronic infection at the large mucosal interface intensifies mitochondrial damage [107, 105]. Impaired mitochondrial function impedes adenosine triphosphate (ATP) synthesis and calcium regulation, both of which are crucial for neuronal activity [110, 111]. In addition, the impact of mitochondria on PD is mediated through its interaction with PD-related proteins. The intricated nature of mitochondria and its involvement in various pathogenic pathways associated with PD underscores its significant influence on several processes, such as energy supply, calcium buffering, and ROS production [111]. For instance, PINK1 actively recruits Parkin to mitochondria and phosphorylates it, leading to the disruption of mitochondria. This disruption allows for their encapsulation by autophagosomes and subsequent transportation to lysosomes [110]. Animal models with PINK1 and Parkin mutations presented structural and functional alterations in mitochondria, which were frequently implicated in the degeneration of DA neurons in these models [100].

A synergistic effect has been identified between the coexistence of *H. pylori* and SIBO with a diminished effectiveness of PD medications and subsequently exacerbating motor fluctuations [100]. *H. pylori* infection was more closely associated with wear phenomena, whereas SIBO was primarily correlated with delayed opening and non-opening. The simultaneous presence of *H. pylori* and SIBO resulted in the most severe motion fluctuations [100]. PD patients with comorbid SIBO experienced more severe dyskinesia, including prolonged rest time, delayed on-time and off-time, compared to those without SIBO [18]. This suggests a close association between SIBO and deterioration in motor function [18]. Another evidence showed that motor fluctuations in PD patients were improved after the eradication of SIBO, further supporting the association between SIBO and motor fluctuations in PD [88]. Several speculations have been proposed to explain this association, including the potential impact of SIBO on drug absorption by altering chyme composition [18]. Malabsorption can be attributed to the loss of brush border disaccharidase activity caused by mucosal damage, bacterial fermentation of sugars, and bacterial uncoupling of bile acids [112, 113]. SIBO may induce detrimental inflammatory effects on enterochromaffin cells and further impede gastric emptying [114]. Recently, given that levodopa is primarily absorbed in the duodenum, SIBO may hinder drug absorption due to intestinal mucosal inflammation [13, 100, 32].

### *H. Pylori*, BBB and PD

**Fig. 2:**
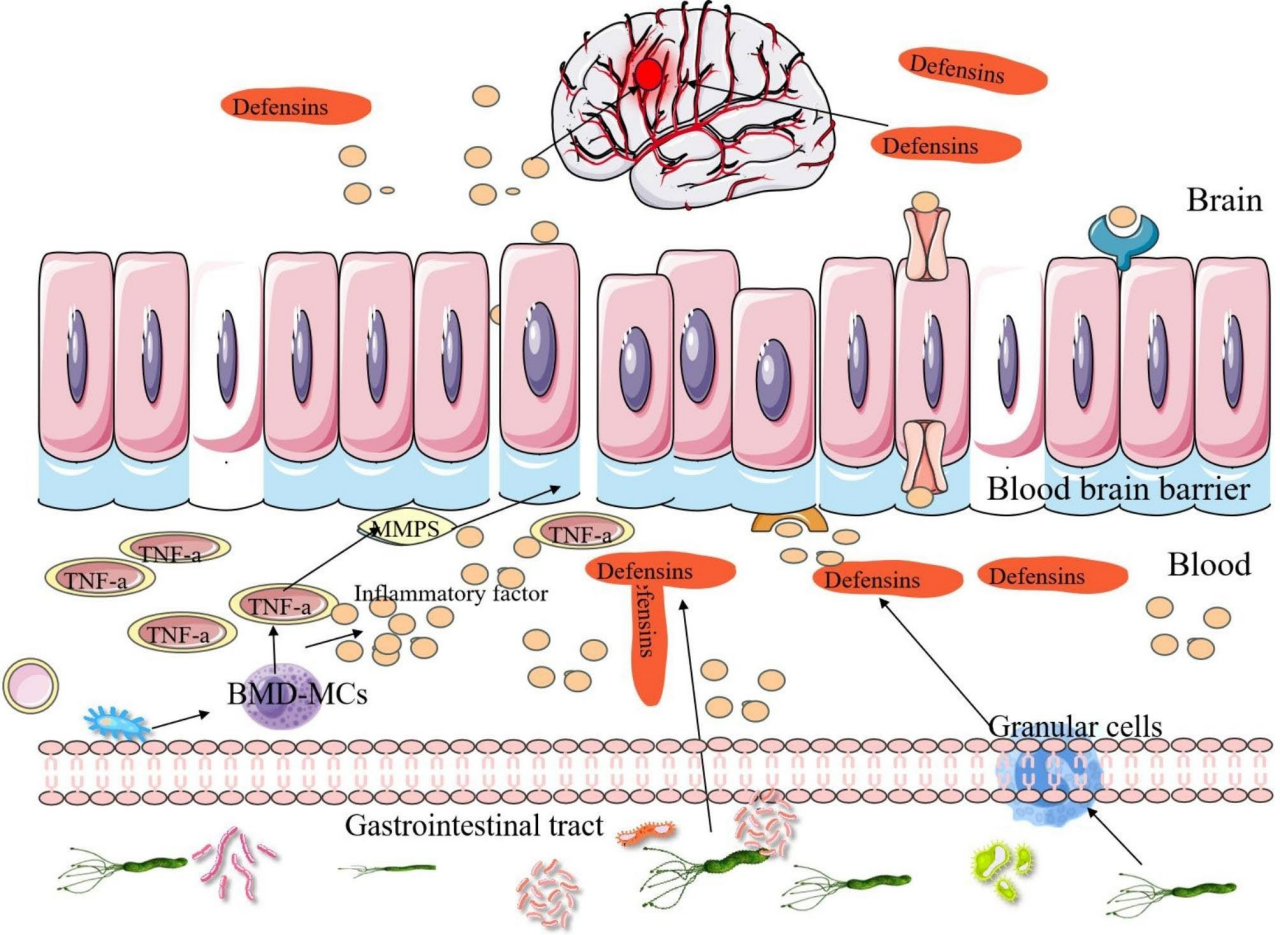
*H. pylori* infection disrupt the BBB to contribute to the development of PD. *H. pylori* infection can stimulate the release of pro-inflammatory cytokines and defensins from BMD-MCs and granulocytes, respectively, resulting in the compromised integrity of the BBB. Consequently, compromised BBB facilitates the infiltration of additional inflammatory factors into the brain, leading to neuronal damage

*H. pylori* has been found to disrupt BBB, leading to an increase of harmful substances entry into the brain and subsequent neuronal damage (Fig. 2) [114]. This disruption is attributed to the promotion of pro-inflammatory cytokines, IFN-γ and TNF-α, release from bone marrow-derived mast cells (BMD-MCs) by *H. pylori* infection, with TNF-α activating matrix metalloproteinases that compromise the integrity of BBB [13]. In addition, *H. pylori* may contribute to BBB disruption through the release of defensins, particularly those that are uniquely distributed at the site of BBB [115]. Simultaneously, *H. pylori* infection triggers the activation of granulocytes and prompts the release of defensins from these cells. The defensins, once secreted by the activated granulocytes, are capable of breaching BBB and gaining access to brain, potentially resulting in neurodegeneration [116]. On the other hand, the inflammatory factors instigated by *H. pylori* can disrupt the neuroendocrine immune system and impair brainstem function, leading to the prolonged secretion of pro-inflammatory cytokines that compromise the integrity of BBB [13]. Consequently, this compromised barrier permits the entry of more detrimental substances into the brain, thereby causing damage to DA neurons and inducing PD.

## *H. pylori* and levodopa bioavailability

**Fig. 3:**
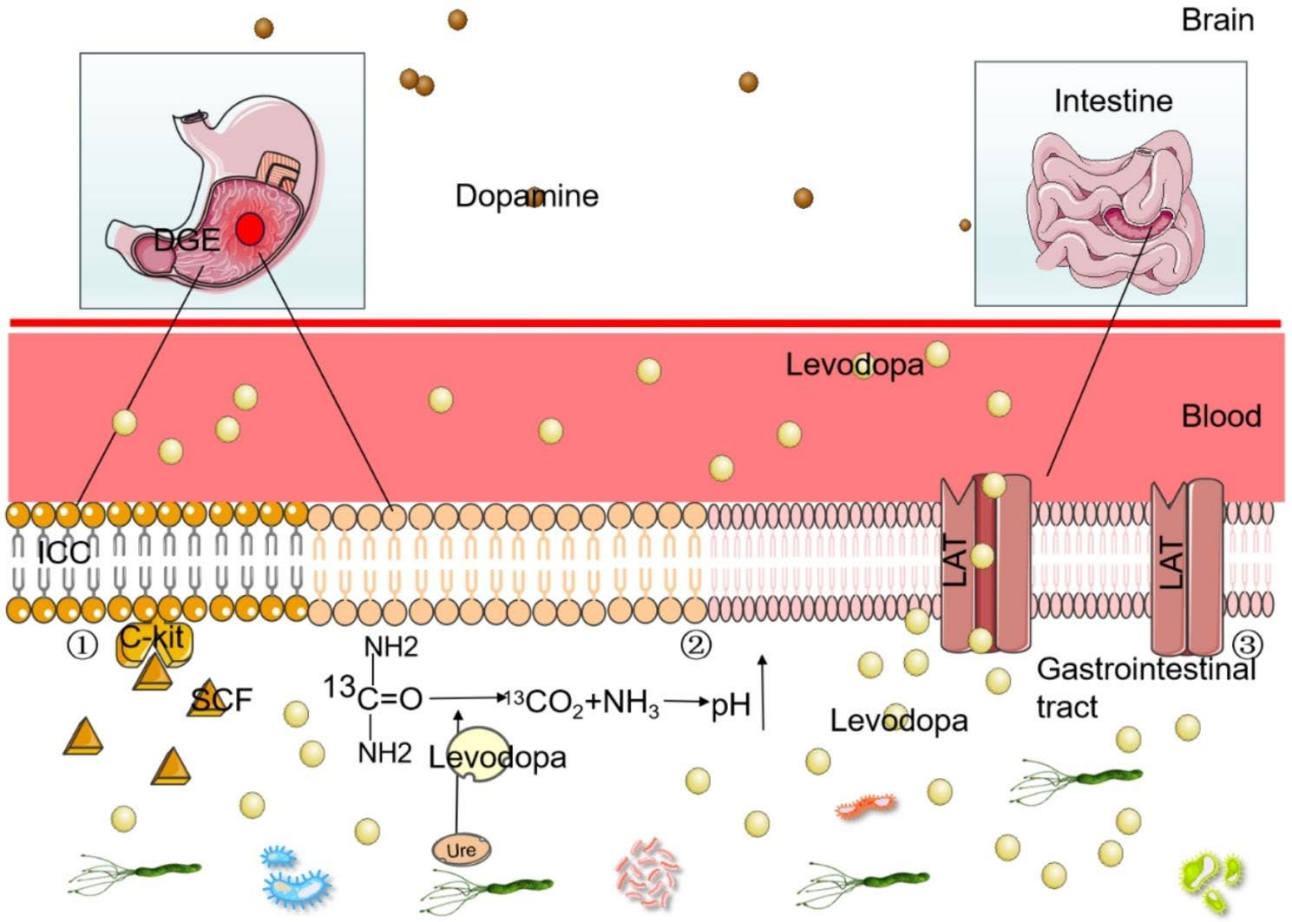
Effect of *H. pylori* infection on levodopa absorption. (1) *H. pylori* infection induces gastroparesis by regulating the SCF/c-kit pathway, hereby further impeding levodopa absorption. (2) *H. pylori* infection obstructs the dissolution of levodopa under elevated pH conditions. (3) *H. pylori* infection leads to duodenal enterocolitis, which hampers levodopa absorption through LATs. Abbreviations: ICC: Interstitial cells of Cajal; SCF: stem cell factor. DGE: Delayed gastric emptying; LAT: L-type amino acid transporter

As previously mentioned, the primary approach to managing PD involves alleviating motor symptoms, with levodopa being a crucial medication for addressing these symptoms [116, 117]. Extensive studies have been conducted in recent years, particularly focusing on the in vivo metabolism of levodopa. It is widely recognized that oral administration of levodopa causes its absorption into the bloodstream at the duodenum and subsequent passage through the BBB to reach the pathological area for therapeutic purposes. Numerous factors influence the bioavailability of levodopa during this process, and among them, *H. pylori* has been found to exert a significant impact on the efficacy of levodopa [118, 119]. *H. pylori* interferes with the efficacy of levodopa treatment, leading to motor fluctuations [120]. This phenomenon has been substantiated by a study conducted in Korea, wherein a notable reduction of 25.9% in levodopa “onset” time and an increase of 11.9% in levodopa “on time” were observed after successful eradication of *H. pylori* infection in 34 patients with PD. Notably, there was no significant alteration in symptom scores, as evaluated by the unified-PD rating scale III, before and after eradication [121]. Consequently, the eradication of *H. pylori* infection could serve as a preventive measure and improve the clinical condition of PD patients experiencing motor fluctuations [34]. The impact of *H. pylori* on levodopa pharmacokinetics in PD is multifaceted (Fig. 3).

### *H. Pylori* and gastroparesis

Pathological alterations of the intragastric milieu during *H. pylori* infection in PD affect the process of drug absorption [34]. Among gastrointestinal disorder in PD, gastroparesis is the most prevalent [122]. In addition, there is evidence suggesting a correlation between the severity of dyskinesia in PD patients and the gastroparesis [9]. Clinically, gastroparesis, also namely delayed gastric emptying (DGE), is characterized by slowing or stopping the food movement from stomach to small intestine, even though there is no blockage in the stomach or intestines, and *H. pylori* infection influences DGE and hampers the absorption of levodopa [118, 34, 123]. The main feature is reduced by gastric motility in the absence of mechanical obstruction [124]. The pathogenic mechanisms of mild paralysis may encompass autonomic and intrinsic neuropathy of the excitatory and inhibitory systems of the gastrointestinal tract, including impairment of the Vagus nerves and interstitial cells of Cajal (ICCs), which govern the contractility of smooth muscle cells [122]. ICCs serve as pacemakers, generating slow waves within the gastrointestinal tract, thereby dictating the frequency of rhythmic contractions in the smooth muscle [125–127]. ICCs are ubiquitously distributed throughout the entire gastrointestinal tract, spanning from the esophagus to the internal anal sphincter [125]. They act as mediators of muscle innervation in motor activity, and the proliferation, differentiation and functionality of ICCs are intricately associated with the activation of c-kit receptors present on their surface [128]. The activation of c-kit is contingent upon the presence of the endogenous ligand, stem cell factor (SCF) [129, 130]. Previous investigations have demonstrated that SCF mutations or deletions precipitate a decrease of ICCs number or disrupt the integrity of the ICC network, thereby giving rise to gastrointestinal motility disorders [131–133]. Alterations in SCF levels can consequently induce changes in the number of ICCs, leading to gastrointestinal motility disorders [134]. Hence, it is evident that the SCF/c-kit pathway plays a crucial role in the proper functioning of ICCs. In addition, increasing studies have demonstrated that *H. pylori* infection affects the SCF level, leading to a reduction in the number of ICCs and subsequently causing DGE. Recent findings conducted in 2021 revealed a significant decrease in gastric emptying rates among *H. pylori*-infected mice compared to uninfected mice [123]. Moreover, the expression of SCF in gastric tissues of *H. pylori* infection was obviously lower compared to the *H. pylori*-uninfected group. Also, submucosa and intramuscular ICCs numbers were reduced in the *H. pylori*-infected group. These findings suggest that *H. pylori* infection is highly associated with a decrease in SCF levels and ICCs numbers in gastric tissue, eventually leading to DGE. Furtherly, the entry of levodopa into the duodenum is affected by this phenomenon [125].

### *H. Pylori* and stomach pH

Influence of stomach pH on levodopa dissolution has been observed, indicating that levodopa exhibits optimal solubility in highly acidic environments [34, 123]. However, the regulation of stomach pH by *H. pylori* complicates this process. Specifically, *H. pylori* produces urease in the stomach, which hydrolyzes urea into ammonia and carbonic acid. The ammonia by-products act as buffers for gastric acid, thereby regulating stomach pH [102, 123]. Consequently, the presence of *H. pylori* hinders the dissolution of the drug in higher pH conditions. In addition, *H. pylori* infection causes chronic inflammation of the gastric mucosa, resulting in the inhibition of gastric acid secretion by the mural cells [121, 135]. Pro-inflammatory cytokine, such as IL-1β, a key mediator in *H. pylori*-associated disease, inhibits gastric acid secretion in vitro and in vivo [136]. Prolonged injury from *H. pylori* could cause an obvious loss of mural cells, leading to atrophy of the oxidative glands and a subsequent decrease in gastric juice acidity [135].

Furthermore, it has been discovered that *H. pylori* possesses several types of receptors that enable it to directly utilize levodopa for growth [118, 39]. The proliferation of *H. pylori* necessitates specific amino acids, including arginine, histidine, isoleucine, leucine, methionine, valine, and phenylalanine. Notably, the demand for phenylalanine presents the potential for bacterial consumption of levodopa within the stomach. Various animal studies have substantiated this assertion [137, 138]. In a speculative study published in 2010, it was indicated that *H. pylori* colony-forming units had a direct reliance on levodopa for their growth [136]. Time- and concentration-dependent incubation studies indicated an apparent decrease in levodopa levels upon contact with *H. pylori* [122]. Moreover, an inverse relationship between the concentration of *H. pylori* and levodopa was observed as time progressed. *H. pylori* exhibited the enhanced growth in media containing higher level of levodopa compared to media simulating the normal gastric environment [138].

### *H. Pylori* and duodenum

Levodopa is primarily absorbed in the duodenum, making it a crucial site for modulating the efficacy of levodopa treatment. Extensive studies have documented the impact of duodenal enterocolitis on levodopa absorption, with multiple studies demonstrating the association between *H. pylori* infection and duodenal enterocolitis [118, 123]. Activation of human phagocytes by *H. pylori* leads to the production of reactive oxygen metabolites (ROM), which inflict damage upon the duodenal mucosa. This process plays an important role in the development of enterocolitis and subsequently affects levodopa absorption [139]. Absorption of levodopa in the small intestine is believed to be facilitated by a significant neutral amino acid transport mechanism, potentially involving the L-type amino acid transporter protein (LATs) [140]. This transporter is closely involved in the transportation of various amino acids. To minimize competition for carrier-mediated intestinal transport, it is advisable to avoid the simultaneous. Once absorbed into the blood stream, levodopa traverses BBB with the assistance of LATs. However, the presence of duodenal inflammation hinders the functional absorption of levodopa by LATs [39].

## Conclusions

Recent studies have identified numerous factors that are associated with the onset and progression of PD, thereby presenting a significant challenge in treatment due to the diversity of these influencing factors. Consequently, approach to treating PD symptoms has not solely focused on addressing the symptoms themselves, but rather encompasses the management of other factors that impact PD. Notably, *H. pylori* alone could exert multiple effects on PD, thereby exacerbating the complexity of treatment. Eradication of *H. pylori* infection might improve clinical status of patients with PD, especially on bradykinesia, or improve levodopa bioavailability. However, some studies pointed out *H. pylori* eradication did not improve clinical outcomes in PD. Therefore, identification and subsequent targeted treatment of the underlying cause of PD have emerged as the paramount concerns and the optimal therapeutic choice presently. Moreover, this simultaneous treatment approach presents several discernible drawbacks. Primarily, the amalgamation of various drugs is likely to result in a diverse range of side effects, thereby detrimentally impacting the patient’s overall health. Secondly, this approach imposes a substantial financial burden on the individual. Finally, the aforementioned factors significantly compromise the physical and psychological well-being of patients.

## Data Availability

Not applicable.

## Abbreviations

α-syn: α-synuclein
ATF-2: Activating transcription factor 2
ATP: Adenosine triphosphate
AD: Alzheimer’s disease
BBE: Bickerstaff brainstem encephalitis
BBB: Blood-brain barrier
BMD-MCs: Bone marrow-derived mast cells
CagA: Cytotoxin-related gene A
CG: Cholesterol glucosides
CGT: Cholesterol-based glycosyltransferase
CNS: Central nervous system
COX-2: Cyclooxygenase-2
DA: Dopamine
DGE: Delayed gastric emptying
ENS: Enteric nervous system
GBS: Green-Barré syndrome
*H. Pylori*: *Helicobacter pylori*
iNOS: Inducible nitric oxide synthase
ICCs: Interstitial cajal cells
LATs: L-type amino acid transporter
LBP: Lipopolysaccharide binding protein
LEs: Late endonuclear bodies
LPS: Lipopolysaccharide
MS: Multiple sclerosis
NADPH: Nicotinamide adenine dinucleotide phosphate
NAP: Neutrophil activating protein
NMR: Nuclear magnetic resonance
NO: Nitric oxide
PD: Parkinson’s disease
ROS: Reactive oxygen species
ROM: Reactive oxygen metabolites
PPIs: Proton pump inhibitors
SCF: Stem cell factor
SIBO: Small intestinal bacterial overgrowth
TLR4: Toll-like receptor 4
TNFR1: Tumor necrosis factor receptor 1
Ure: Urease
VacA: Vacuolar cytotoxin A
